# Whole genome sequencing for tuberculosis disease species identification, lineage determination, and drug resistance detection in Kashgar prefecture, China

**DOI:** 10.1186/s12879-025-11221-w

**Published:** 2025-10-07

**Authors:** Dongxin Liu, Gulina Badeerhan, Mawlanjan Emam, Mengnan Jiang, Geng Hong, Mengjiao Xie, Yang Liu, Ling Ma, Lin Xu, Xijiang Wang, Qiang Wei

**Affiliations:** 1 Chinese Center for Disease Control and Prevention, National Pathogen Resource Center, Beijing, China; 2 Xinjiang Uighur Autonomous Region Center for Disease Control and Prevention, Urumchi, China; 3 Kashgar District Center for Disease Control and Prevention, Kashgar, China; 4 Gansu Provincial Center for Disease Control and Prevention, Lanzhou, China; 5 Yunnan Provincial Center for Disease Control and Prevention, Kunming, China

**Keywords:** Tuberculosis, WGS, Drug-resistant, Species identification, Lineage

## Abstract

**Background:**

We aimed to use whole genome sequencing (WGS) to determine species and lineage composition and drug resistance profile in a high tuberculosis (TB)-burden region of China.

**Methods:**

We conducted WGS to 1791 acid-fast staining positive and culture-positive isolates collected from Kashgar prefecture in 2020. Bioinformatic analysis was applied to confirm species, lineage and drug resistant-related mutations. The drug susceptibility testing was performed on confirmed *Mycobacterium tuberculosis* complex (MTBC) isolates. We determined the accuracy of WGS prediction by comparing with phenotypes.

**Results:**

95.03% (1702/1791) were identified MTBC, 3.18% (57/1791) were *nontuberculous mycobacteria* (NTM), 0.61% (11/1791) were nocardia, 0.89% (16/1791) were gordonia and 0.056% (1/1791) were rhodococcus, the rest 4 isolations were identified as mixed infection. MTBC were composed of lineage 2 (45.83%, 780/1702), lineage 3 (462/1702, 27.14%), lineage 4 (455/1702, 26.73%), lineage 1(1/1702, 0.06%) and *M.bovis* (La1, 4/1702, 0.24%). Resistance to rifampicin, ethambutol, fluoroquinolones, aminoglycosides and ethionamide were accurately predicted with sensitivity of 96.43%, 83.33%,100%, 100% and 94.74% by WGS, while resistance to isoniazid with the sensitivity of 81.62%.

**Conclusions:**

WGS can be an important approach in assessing TB control strategy and for determining therapeutic schemes in high TB-burden regions. The drug resistance TB of Kashgar prefecture is at low level and the application of WGS may prevent the increase of resistance rate.

**Supplementary Information:**

The online version contains supplementary material available at 10.1186/s12879-025-11221-w.

## Introduction

Tuberculosis (TB) remain a leading cause of death worldwide with 10.6 million new cases and 1.3 million death cases in 2022 globally. China has the third largest TB burden, which there were an estimated 748,000 new TB cases in 2022, accounting for 7.1% of TB incident cases worldwide [1]. Kashgar prefecture in Xinjiang Autonomous Region is one of the areas with the highest tuberculosis incidence rate in China with a incidence of 806.75 cases per 100,000 people in 2018 [2]. Moreover, as Kashgar prefecture is located in an important hub of the Silk Road and borders Pakistan, India, and other countries, *Mycobacterium tuberculosis* (*Mtb*) may be introduced into the region along with population migration, trade and cultural exchange [3]. Kashgar has therefore become the critical region in the context of TB control in China.

To formulate the targeted strategies of TB prevention and control in Kashgar, the starting point is to have scientific information associated with species identification, *Mtb* strain lineage distribution and drug resistance characteristics in this region. Unfortunately, the above information remains ambiguous due to the insufficient species diagnosis accuracy of conventional Ziehl–Neelsen (ZN) smear microscopy [4], and high cost of phenotypic drug susceptibility testing [5]. Molecular platforms such as Xpert MTB/RIF Ultra have delivered the capacity to confirm the presence of *Mtb* and resistance to rifampin from sputum sample [5, 6], nevertheless, as such targeted molecular assays are vulnerable to off-target emerging mutations and provide limited information on susceptibility to other drugs or identification of other strains. Treatment for many patients remains semi-empirical, with an increased risk of treatment failure and amplification of resistance to more drugs [7].

Whole-genome sequencing (WGS) can identify species and lineage, predict drug susceptibility with high accuracy through identifying specific relevant mutations and has been heralded as a potential solution for the implementation of continuous surveillance and personalized therapy [8, 9]. In principle, WGS provides the ultimate resolution for strain classification to trace infection sources and transmission networks as well as allows for the simultaneous prediction of the complete antimicrobial susceptibility profile of a given isolate.

The current study aimed to accurately identify the strains species and circulating *Mtb* lineages and determine their drug resistance characteristics among TB cases in one of the highest TB-burden regions in China using WGS and to provide scientific basis to foster targeted TB control strategies.

## Methods

### Sample source and sample collection

Kashgar prefecture lies in the southwest of Xinjiang province and consists of 11 counties and one county-level city (Supplementary Fig. 1), and has consistently been amongst the regions burdened by a high prevalence of TB in China. In 2020, the TB case notification rate in Kashgar was 250.74 per 100,000 people. Kashgar CDC conducted this survey and collect sputum samples and clinical information for eligible individuals. Eligible patients were those presenting as presumptive tuberculosis cases newly registered during the survey period (January 2020 to December 2020) at selected tuberculosis clinics. A presumptive case was defined as a patient with a persistent cough for more than 2 weeks or at least two of the following symptoms: fever, drenching night sweats, unexplained weight loss (> 1·5 kg/month), general feeling of illness (malaise) and tiredness, and shortness of breath with chest pain [10]. This study was approved by the Tuberculosis Research Ethics Review Committee of the Kashgar CDC, and written informed consent was obtained from each participant. All authors vouched for the completeness and accuracy of the data presented.

### Specimen processing and sequencing

Two sputum samples for culturing were obtained from each eligible patient who can provide qualified sputum before the initiation of treatment. To isolate the culture, each specimen was treated with 4% sodium hydroxide, followed by homogenization through vigorous stirring. Subsequently, an aliquot of 0.1 ml from the resulting specimen was inoculated into two tubes of acidified Löwenstein–Jensen medium, and the inoculated tubes were then incubated at 37 °C. The cultures were assessed during the first week for rapidly growing bacteria and subsequently every week thereafter for slower growing bacteria. If no bacteria were detected by the eighth weeks, the result was recorded as negative [11, 12]. All culture-positive strains were sent to Kashgar CDC for nucleic acid extraction and phenotypic drug susceptibility testing.

### Drug susceptibility testing

Drug susceptibility testing was performed using the UKMYC6 96-well microdilution plate format, designed by the CRyPTIC project (Thermo Fisher Inc., UK). The UKMYC6 plate allowed for the determination of susceptibility against 13 antituberculosis drugs composed of agents used in drug-susceptible TB treatment (rifampicin, rifabutin, and isoniazid) as well as longer MDR-TB treatment corresponding to groups A (levofloxacin, moxifloxacin, bedaquiline, and linezolid), B (clofazimine), C (ethambutol, delamanid, and amikacin, ethionamide) and kanamycin [10]. The ECOFF for each drug were determined according to Philip’s research work [13].

### Whole genome sequencing and bioinformatics analysis

Nucleic acid was extracted using the cetyl trimethyl ammonium bromide method. Genomic DNA was sequenced using the Illumina Hiseq-2000 with an expected depth of 200x. To identify the species, each dataset of sequencing reads was analyzed by Kraken tool (v2.1.1) [14]. If MTBC accounted for less than 70% of a sample, the sample were identified as mixed infection with other species and excluded for following analysis [15]. For those identified as MTBC, paired-end reads were aligned to the reference genome H37Rv (NC_000962·3) using the Burrows-Wheeler algorithm and sorted with SAMtools (V.1.15). Variant calling was conducted with GATK to identify single nucleotide polymorphisms, with low-quality SNPs (Phred score Q < 20 and read depth < 5) and sites with missing calls in > 10% of isolates removed from analysis. SNPs located within 12 bp of each other or that had less than 75% of supporting high quality reads were also excluded. Variations in known drug-resistant genes or intergenic regions, direct repeat regions, microsatellite like sequences, transposition insertion sequences (such as IS6110), ESX secretion system protein genes, and PE/PPE/PGRS family genes were masked for phylogenetic tree reconstruction [12, 16, 17]. A maximum likelihood (ML) phylogeny was inferred using RaxML-NG v1.0.3, and the substitution model was GTR + G [18]. In silico drug resistance prediction was carried out using TBProfiler [19].

### Statistical analysis

Drug resistance rates are expressed as numbers (percentages). Multivariable logistic regression models were used to examine the odds ratios (ORs) and 95% confidence intervals (CIs) for the risk factors that were associated with drug resistance rate. Bar charts were used to display the percentage composition of MIC for various drugs. A pie chart was used to display the composition of bacterial types. The phenotypes were used as the gold standard to calculate the sensitivity and specificity of gene detection method. A 2-sided *P* value < 0.05 was used to determine statistical significance. Data cleaning and statistical analyses were performed using SAS version 9.2 (SAS Institute).

## Results

### Strain data description

**Fig. 1:**
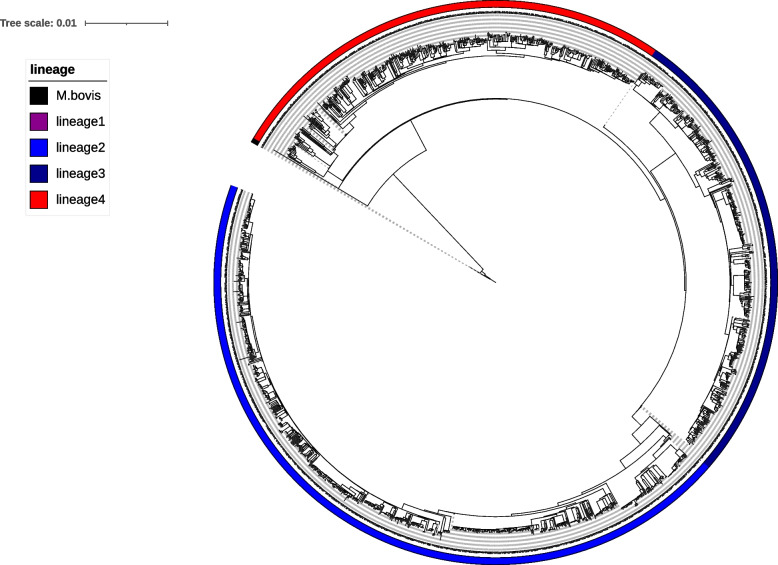
Phylogeny of epidemics M. tuberculosis complex strains in Kashgar Prefecture 1700 MTBC isolates and H37Rv were used for phylogenetic tree construction. The tree is drawn to scale, with branch lengths measured in the number of substitutions per site

The Kashgar Tuberculosis Survey 2020 collected 1972 bacterial strains samples from 12 selected sites of all Kashgar prefecture. Cultures of specimens from 181 patients failed to recover or were contaminated. Among the 1791 revived strains, based on Kraken analysis, 85 acid-fast staining samples were identified to be from NTM (57 isolates), nocardia species (11 isolates), gordonia species (16 isolates) and rhodococcus species (1 isolates); 1702 isolates were identified as mycobacterium tuberculosis complex (Table 1 and Supplementary Fig. 2); and 4 were identified as mixed infection. Five lineages were observed through the WGS analysis. Phylogenetic analysis showed that the majority coming from L2 (780 isolates; 45.83%), L3 (462 isolates; 27.14%) and L4 (455 isolates; 26.73%), while L1 (1 isolates; 0.06%) and *M. bovis* (4 isolates; 0.24%) comprised the remaining ones (Fig. 1).

**Table 1:** Composition of acid-fast staining positive strains in Kashi prefecture

Species	Isolates number	Percentage(%)
*Nocardia cyriacigeorgica*	8	0.45
*Nocardia farcinica*	3	0.17
*Rhodococcus pyridinivorans*	1	0.06
*Gordonia bronchialis*	2	0.11
*Gordonia polyisoprenivorans*	12	0.67
*Gordonia sp. YC-JH1*	1	0.06
*Gordonia terrae*	1	0.06
*Mycobacterium intracellulare*	1	0.06
*Mycobacterium novum*	3	0.17
*Mycobacterium sp. DL440*	5	0.28
*Mycobacterium sp. DL90*	2	0.11
*Mycobacteroides chelonae*	5	0.28
*Mycolicibacillus koreensis*	4	0.22
*Mycolicibacter minnesotensis*	2	0.11
*Mycolicibacterium boenickei*	9	0.50
*Mycolicibacterium celeriflavumavum*	2	0.11
*Mycolicibacterium fortuitum*	1	0.06
*Mycolicibacterium mageritense*	1	0.06
*Mycolicibacterium phlei*	1	0.06
*Mycolicibacterium pulveris*	18	1.01
*Mycolicibacterium thermoresistibile*	3	0.17
*Mycobacterium tuberculosis complex*	1702	95.03
*Mycobacterium tuberculosis* mixed infection	4	0.22
Total	1791	100%

### Drug-resistant profile

Phenotypic drug susceptibility results were obtained for 1664 isolates. The drug with highest resistance rate was isoniazid (9.07%, 151/1664), and the second was rifampin (3.25%, 54/1664). The MDR rate was 2.4% among Kashgar region. The resistant rate for the detailed resistance profile of all the drugs are showed in Table 2.

**Table 2:** Drug resistant prevalence of Mtb for 13 drugs among Kashgar cases

Drugs	Total number	Durg resistance number	Resistance rate(%)
Bedaquiline	1664	7	0.42
Clofazimine	1664	12	0.72
Delamanid	1664	10	0.60
Ethambutol	1664	5	0.30
Ethionamide	1664	18	1.08
Rifabutin	1664	44	2.64
Linezolid	1664	0	0
Rifampicin	1664	54	3.25
Kanamycin	1664	4	0.24
Levofloxacin	1664	18	1.08
Isoniazid	1664	151	9.07
Moxifloxacin	1664	14	0.84
Amikacin	1664	4	0.24
Multiple-drug resistant	1664	40	2.40

**Fig. 2:**
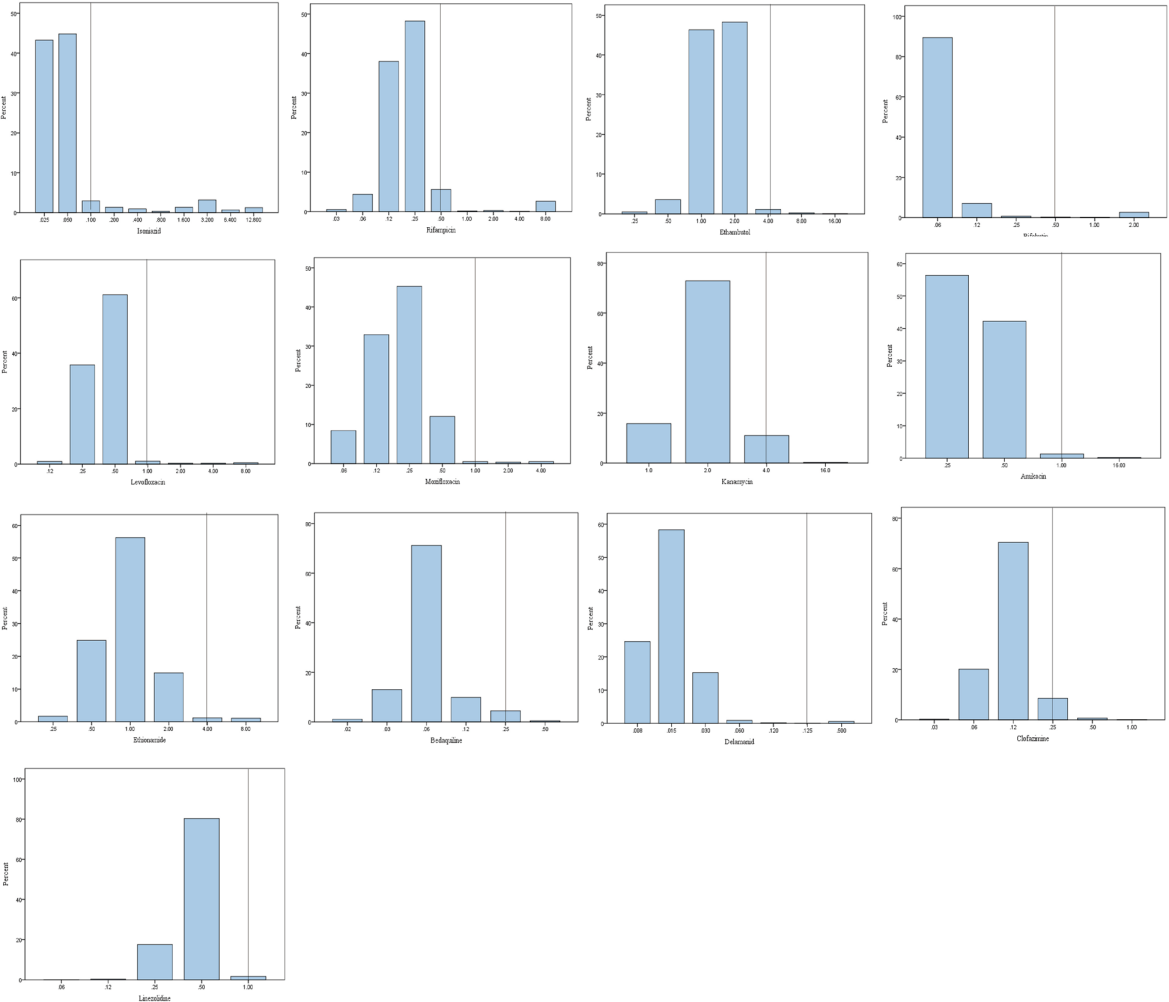
The overall MIC distributions against 13 anti-TB drugs MIC: minimal inhibitory concentration; Dotted line represent ECOFF

Different from the agar proportion method critical concentrations, MIC susceptibility testing method can provide quantitative results, the overall MIC distributions for 13 drugs are showed in Fig. 2. We discovered that although the drug resistance rate is at low level, there are many strains with MIC at ECOFF levels, such as, 49 isolates (2.94%, 49/1664) showing MIC value of isoniazid at 0.1 μg/mL, 93 isolates (5.59%, 93/1664) having MIC value of rifampin at 0.5 μg/mL, and 18 isolates (1.08%) showing MIC value of levofloxacin at 1 μg/mL.

### Prediction efficiency of WGS

The WGS prediction demonstrated sensitivities of 81.62%, 95.65%, and 83.33% for first-line anti-TB drugs isoniazid, rifampin, and ethambutol, respectively, with specificities of 98.82%, 99.44%, and 99.1%. Sensitivities for second-line drugs ranged from 94.74% to 100% (Table 3). Due to low drug resistant rate and predictive sensitivity, prediction accuracy were not analyzed for new anti-TB drugs (bedaquiline and delamanid) or repurposed drugs (clofazimine and linezolid).

**Table 3:** Prediction of phenotypes of resistance to nine drugs by WGS

Drug	Resistant phenotype			Susceptible phenotype			Sensitivity(%)	Specificity(%)
	R	S	total	R	S	total		
Isoniazid	117	34	151	18	1495	1513	81.62	98.82
Rifampicin	52	2	54	9	1601	1610	96.43	99.44
Rifabutin	42	2	44	19	1601	1620	95.65	98.84
Ethambutol	4	1	5	15	1644	1659	83.33	99.1
Amikacin	4	0	4	2	1657	1659	100	100
Kanamycin	4	0	4	2	1657	1659	100	100
Ethionamide	17	1	18	19	1625	1644	94.74	98.86
Levofloxacin	18	0	18	1	1645	1646	100	100
Moxifloxacin	14	0	14	5	1645	1650	100	100

*R* Resistant, *S* Susceptible

## Discussion

Kashgar has always been one of the highest TB-burden regions in China. In 2020, the reported TB incidence in Kashgar was 250.74 per 100,000 people, which was 4.3 times as that of the national average for the same time period [2]. Here, we firstly used WGS combined with high-throughput quantitative MIC measurements to determine the species composition, lineage distribution and drug-resistance profile of tuberculosis across the whole Kashgar prefecture.

In recent decades, increasing incidences and prevalences of non-tuberculous mycobacteria (NTM) and Nocard's bacillus have been reported [20], which are widely distributed in the environment and can cause clinical symptoms similar to pulmonary tuberculosis and cause positive results in acid-fast staining, but the drug regimen of them is greatly different from that of TB [21]. Our results showed that as high as 4.75% of the strains (NTM or other acid-fast staining positive species) were misidentified as *Mtb* in Kashgar region based on smear result. The high prevalence of NTM or nocardia or gordonia among symptomatic participants found in this population-based study demonstrates that acid-fast positive bacteria other than *Mtb* has also been a public health problem in this region. As smears are commonly used to diagnose TB and to initiate treatment in Kashgar region, symptomatic NTM or nocardia or gordonia cases seeking care in health facilities could be misdiagnosed and then treated as TB cases. Without adequate clinical response, these cases may subsequently be misdiagnosed as TB treatment failures. Thus, false *Mtb*-positives may in fact be clinical NTM or nocardia or gordonia and if the treatment outcomes do not improve, the patient may mistakenly be classified as DR-TB. Such patient misidentification may negatively impact the health status of the individual and consequently pose additional cost to the health system. Therefore, precise molecular-based species identification methods were urgently needed.

Due to the special geographical location, the MTBC in Kashgar prefecture owe special lineage composition. 1702 cases of MTBC clinical strains were composed of L1-L4 and La1. Lineage 2 was still dominant in Kashgar but its proportion was relatively low compared with other regions of China (45.83% VS 73.9%) [22]. Lineage 3 in Kashgar prefecture accounted for 27.14%, much higher than that of other regions of China, as Kashgar prefecture borders Pakistan (Lineage 3 was dominant; 70.40%) [23], and the border crossing (Khunjerab Port) is located there. It is speculated that the frequent movements of people between the two places caused the spread of Lineage 3 strains to this region [3]. Furthermore, the transmission probability may increase with the growth of population migration, trade, and cultural exchange between the countries. *M. bovis* accounts for 0.24% of all lineages, a much higher proportion than in other regions of China [22, 24]. This may be due to the pastoral lifestyle in Kashgar, where bovine tuberculosis is prevalent in major livestock-producing regions. The zoonotic *M. bovis* strain is often transmitted to humans through contaminated dairy products, leading to lymphatic TB and pulmonary TB [25]. Different *Mtb* lineage is clinically, virulency, and radiologically diverse [26, 27], lineage accurate identification can be most efficiently by using WGS.

It is notable that the drug resistance rate in Kashgar were very low compared with other regions of China [28, 29], the prevalence of MDR tuberculosis was only 2.4%. Drug-resistant TB arises for two reasons: first, the selection of de novo resistance during treatment of the index case, and second, the transmission of drug-resistant TB. Kendall et al. have demonstrated current estimates of MDR-TB prevalence among TB notifications are most consistent with the hypothesis that over 80% of incident MDR-TB in present-day epidemic settings results from transmission of MDR-TB [30]. We believe the main reason for this situation is that all confirmed active TB patients in Kashgar prefecture were hospitalized until the result of sputum smear was negative and then took medication directly observed by village doctor every day after discharge, which reduced the incidence of drug-resistant tuberculosis due to both inappropriate treatment and transmission. This measure should been extended nationwide. In addition, we find new anti-TB drugs (bedaquiline and delamanid) and repurposed drugs (clofazimine and linezolidine) resistant rate was very low as well, which indicates application of a short-course drug-resistant tuberculosis treatment regimen containing new drugs in Kashgar prefecture is promising.

Although Kashgar has low drug resistance rate, it remains at risk of an epidemic of drug-resistant TB. This survey not only highlights the prevalence of drug-resistant TB but also presents the MIC distributions for 13 drugs, as well as the proportion of strains exhibiting MIC values at both the ECOFF and sub-ECOFF resistance levels. These sub-threshold elevations in MIC may still hold clinical significance, given that a combination of substantial interpatient pharmacokinetic variability and elevated MICs can predispose *Mtb* strains to develop resistance, ultimately increasing the risk of treatment failure and adversely affecting patient outcomes [31]. In addition, due to the limitation of laboratory conditions and the lack of skilled personnel, drug sensitivity test cannot be carried out in the whole Kashgar prefecture to detect drug-resistant tuberculosis in time. The existing drug-resistant TB is prone to epidemics easily through transmission. Using WGS combined with quantitative MIC measurements, our results showed that WGS is highly accurate at predicting phenotypic drug resistance except isoniazid in Kashgar prefecture, in consideration of high concordance rates of isoniazid prediction demonstrated by previous studies [16], we speculate that there may be a new isoniazid resistance mechanism of *Mtb* isolates in Kashgar area, which needs further exploration. *Mtb* strains that are resistant to new and repurposed anti-TB agents are rare to date, especially in low TB incidence countries. Consequently, there are insufficient phenotypic DST data globally to confidently interpret mutations associated with resistance to these agents as evidenced by the current WHO catalogue of mutations for *Mtb*, and the accuracy of new and repurposed anti-TB drugs will also improve as data accumulates. Therefore, WGS with the ability to accurately predict drug resistance could give us a more accurate picture of local resistance profile and provide the basis for the formulation of individual patient precision therapeutic regimen. Moreover, with the expansion of commercial sequencing services and sequencing sample sizes, the cost of WGS has decreased to a level lower than that of phenotypic testing and continues to decline, besides, in contrast to the intricate procedures required for phenotypic testing, laboratory personnel need only extract nucleic acids. For these reasons, it could be particularly valuable in settings with weak laboratory capacity and a shortage of skilled people.

Our study had some limitations. First, different resistant mutations can confer different resistant level, but due to the small number of resistant strains, we did not analyze the resistant level of different mutations. Second, due to incomplete information collection and low number of drug-resistant isolates, we did not perform drug-resistant related risk factor analysis.

In conclusion, accurate strain identification, lineage determination and drug resistance detection are very important for TB treatment and control. Our findings prove that with the application of commercial centralized sequencing and automated bioinformatics analysis platforms WGS can have an important role in species and lineage identification, and drug resistance tuberculosis diagnosis, especially in consideration of the limitation of laboratory equipment conditions and the lack of skilled personnel in high TB-burden regions. Additionally, from this study, we have a baseline of tuberculosis drug-resistance mutations in the whole Kashgar prefecture. According to the change in drug-resistance trends in this region, the targeted strategies for drug-resistant TB can be formulated.

## Supplementary Information

Supplementary Material 1. Supplementary Figure 1 Geographical location and counties distribution of Kashgar.

Supplementary Material 2. Supplementary Figure 2 Composition of acid-fast positive strains in Kashgar Prefecture.

## Data Availability

All sequence data is deposited in China National Microbiology Data Center (NMDC) with accession numbers NMDC10018971.

## Abbreviations

TB: Tuberculosis
WGS: Whole-genome sequencing
MTBC: *Mycobacterium tuberculosis* Complex
ZN: Ziehl–Neelsen
ML: Maximum likelihood
ORs: Odds ratios
CIs: Confidence intervals
NTM: Non-tuberculous mycobacteria
