# Supplementary figures and images for: Whole genome sequencing for tuberculosis disease species identification, lineage determination, and drug resistance detection in Kashgar prefecture, China
Source: BMC Infect Dis. 2025 Oct 7;25:1239. doi: 10.1186/s12879-025-11221-w (PMC12502365; doi:10.1186/s12879-025-11221-w)

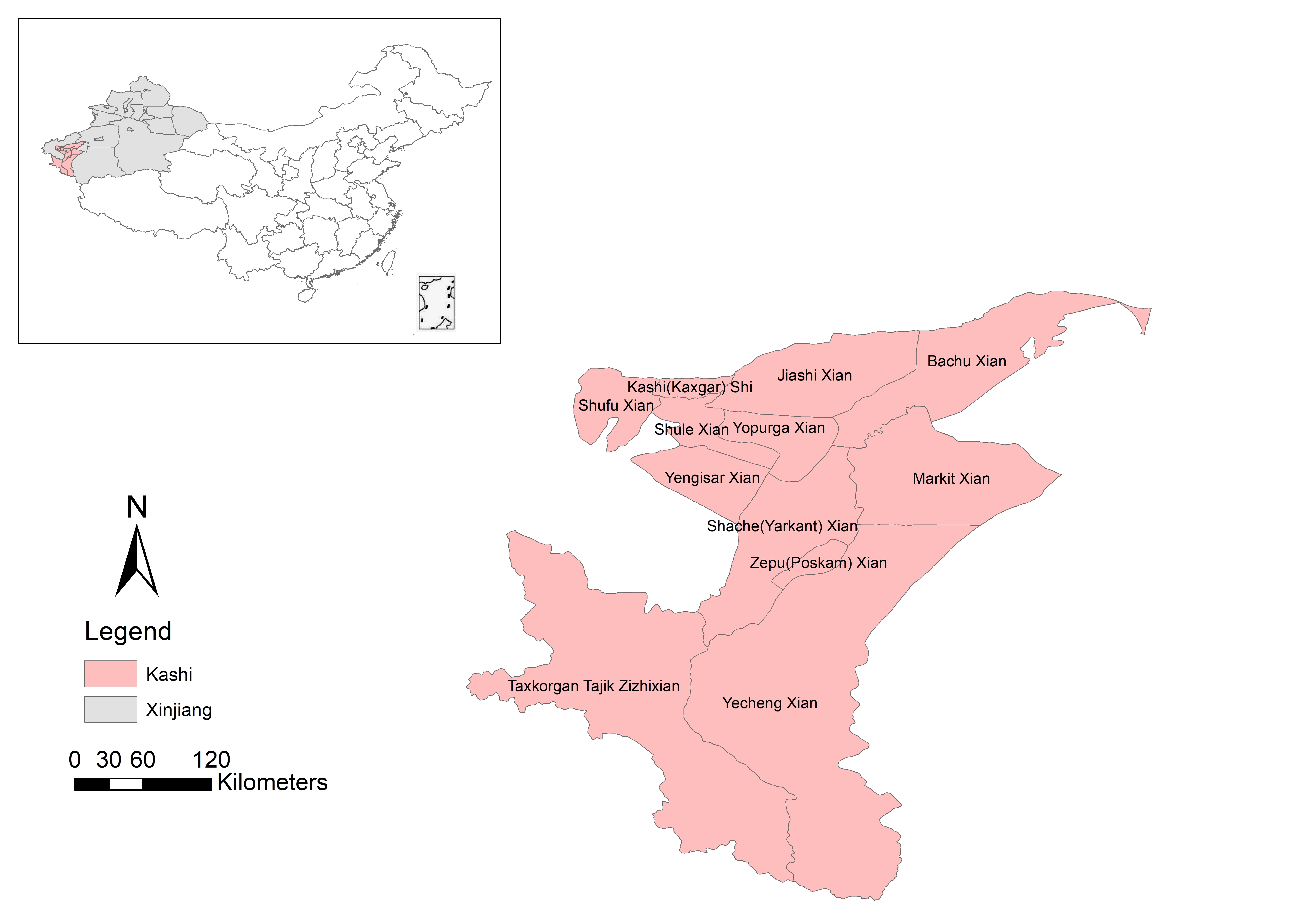

Supplement: Supplementary file 1 — Supplementary Material 1. Supplementary Figure 1 Geographical location and counties distribution of Kashgar. [file 12879_2025_11221_MOESM1_ESM.jpg]

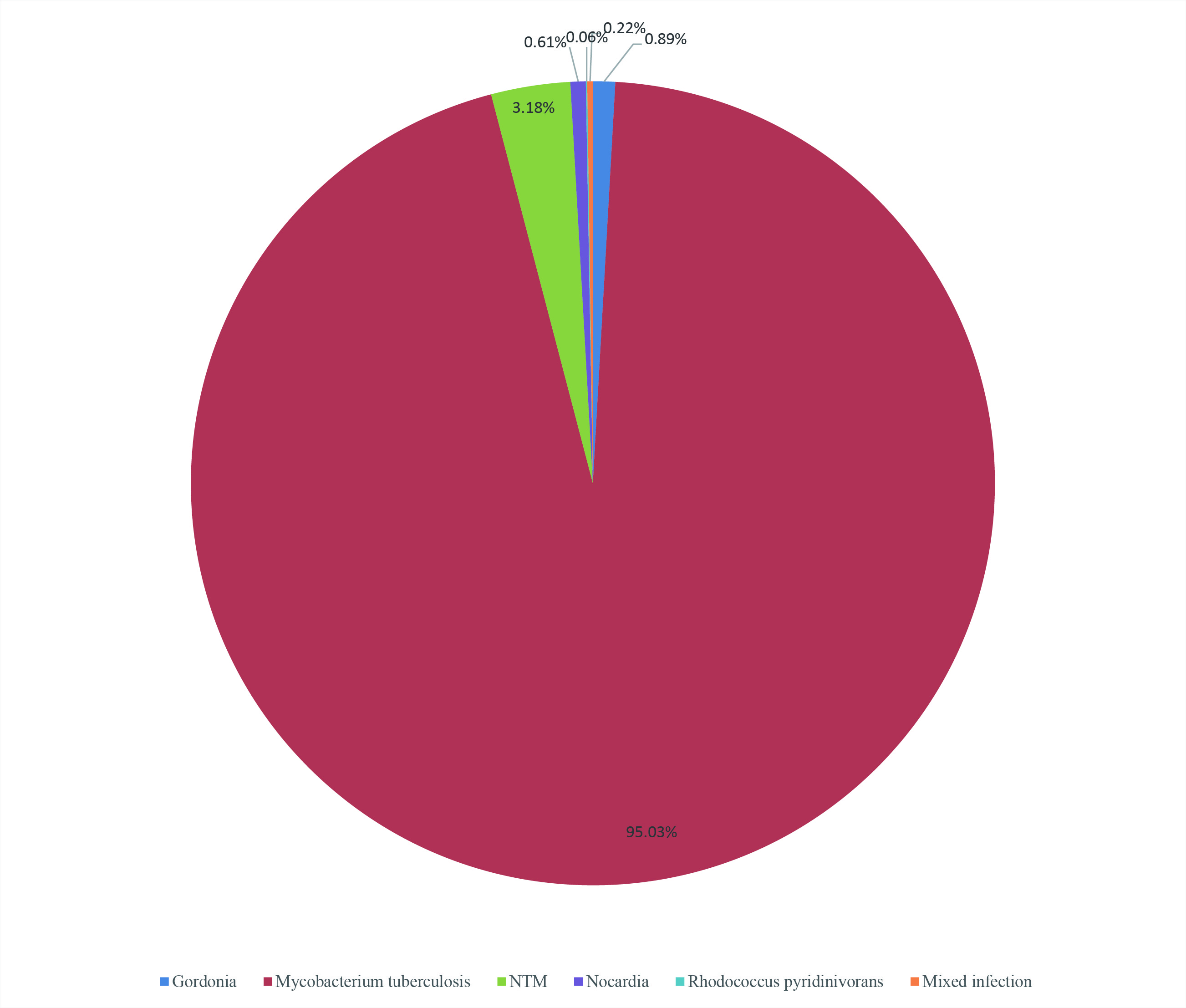

Supplement: Supplementary file 2 — Supplementary Material 2. Supplementary Figure 2 Composition of acid-fast positive strains in Kashgar Prefecture. [file 12879_2025_11221_MOESM2_ESM.jpg]
